# High-Flow Nasal Cannula in Adult Acute Care: Physiological Mechanisms, Clinical Evidence, and Persistent Misconceptions

**DOI:** 10.3390/medicina62101981

**Published:** 2026-10-14

**Authors:** Daniele Orso, Francesco Meroi, Alessandro Brussa, Miriam Ladu, Ornella Spagnolello, Irene Comisso, Nicola Federici, Giorgio Della Rocca

**Affiliations:** 1 Department of Anesthesia and Intensive Care Medicine, “Santa Maria della Misericordia”, Udine University Hospital, Azienda Sanitaria Universitaria Friuli Centrale (ASUFC), 33100 Udine, Italy; francesco.meroi@asufc.sanita.fvg.it (F.M.);; 2 Department of Medicine (DMED), University of Udine, 33100 Udine, Italy

**Keywords:** high-flow nasal cannula, HFNC, respiratory failure, oxygen therapy, non-invasive respiratory support, physiology

## Abstract

High-flow nasal cannula (HFNC) oxygen therapy is widely used in emergency, intensive care, perioperative, and procedural settings, but it is often conceptually assimilated to non-invasive positive-pressure ventilation (NIPPV). This interpretation is physiologically misleading. HFNC is an open high-flow oxygen system whose main effects derive from dead-space washout, improved stability of inspired oxygen fraction (FiO_2_), heated humidification, patient comfort, and only modest flow-dependent positive airway pressure. Its open interface also facilitates eating, drinking, speaking, and secretion clearance. Clinical evidence supports HFNC mainly in selected patients with acute hypoxemic respiratory failure, where it may reduce intubation compared with conventional oxygen therapy. However, evidence is more heterogeneous in hypercapnic respiratory failure, cardiogenic pulmonary edema, immunocompromised patients, obesity, and settings requiring substantial pressure support or ventilatory unloading. NIPPV remains preferred in acidotic hypercapnic COPD exacerbations, while CPAP or NIPPV is indicated in selected pressure-responsive conditions such as cardiogenic pulmonary edema. Persistent excessive effort or clinical deterioration requires timely assessment for invasive ventilation. HFNC should therefore be regarded as optimized high-flow oxygen therapy rather than as “non-invasive ventilation through nasal prongs.” Recognizing this distinction may improve patient selection, guide escalation, and avoid overinterpretation of its clinical effects.

## 1. Introduction

High-flow nasal cannula (HFNC) oxygen therapy has progressively moved from a niche respiratory support technique to a widely used intervention in adult acute care [1]. It is now applied in emergency departments (EDs), intensive care units (ICUs), operating rooms, post-extubation pathways, peri-intubation management, and procedural sedation [2,3,4]. The appeal of HFNC is intuitive: it is easy to apply, generally well tolerated, and permits communication, oral intake, and secretion clearance while providing heated and humidified gas at high flow rates [5]. In the teaching material underlying this review, the device is presented as a system composed of a gas blender, flowmeter, humidifier, circuit, and nasal cannula or tracheostomy connector, with flows up to 60 L/min, fraction of inspired oxygen (FiO_2_) from 0.21 to 1, and gas heated and humidified to near-physiological conditions.

HFNC, non-invasive positive-pressure ventilation (NIPPV), continuous positive airway pressure (CPAP), and conventional oxygen therapy can appropriately be described under the broad umbrella of non-invasive respiratory support (NIRS). Respiratory support encompasses interventions that improve oxygenation, ventilation, or both; it does not imply that all modalities provide mechanical ventilatory assistance [6]. Within this category, NIPPV provides inspiratory pressure support and expiratory positive pressure, whereas CPAP provides continuous distending pressure without a separate inspiratory assist. HFNC is an open high-flow oxygen system acting through anatomical dead-space washout, more stable FiO_2_ delivery, humidification, and modest, variable positive airway pressure. The umbrella term is therefore useful provided these mechanisms and therapeutic indications remain clearly distinguished.

This distinction matters because the clinical interpretation of HFNC is often overstated [7]. Improvement in oxygen saturation may be mistaken for resolution of respiratory failure. Variable upper-airway pressure may be assumed to provide reliably controlled positive end-expiratory pressure (PEEP) equivalent to CPAP or NIPPV [8]. Reduced intubation rates compared with conventional oxygen therapy may be interpreted as evidence of equivalence to NIPPV [9]. These assumptions are physiologically problematic and may become clinically harmful if HFNC delays escalation in patients who require true ventilatory assistance.

The aim of this narrative review is to provide a physiology-oriented interpretation of HFNC in adult acute care. We discuss the main physiological mechanisms, summarize key supporting studies, integrate recent clinical evidence across major indications, and highlight persistent misconceptions that may affect bedside use.

## 2. Technical Characteristics of HFNC

HFNC systems deliver a heated and humidified gas mixture through large-bore nasal cannulas [10,11]. The clinician can independently set gas flow and FiO_2_, allowing a combination of high inspiratory flow delivery and controlled oxygen concentration. This differs from conventional low-flow oxygen devices, in which the effective FiO_2_ depends heavily on patient inspiratory flow, breathing pattern, minute ventilation, and room-air entrainment [10,11].

The technical ability to provide high flow is central to HFNC physiology [10,11]. When delivered flow approaches or exceeds patient inspiratory demand, entrainment of ambient air is reduced and the selected FiO_2_ more closely approximates the inspired FiO_2_. This mechanism is particularly relevant in tachypneic patients, whose peak inspiratory flow may far exceed the flow provided by standard nasal cannulas or simple face masks [10].

**Figure 1:**
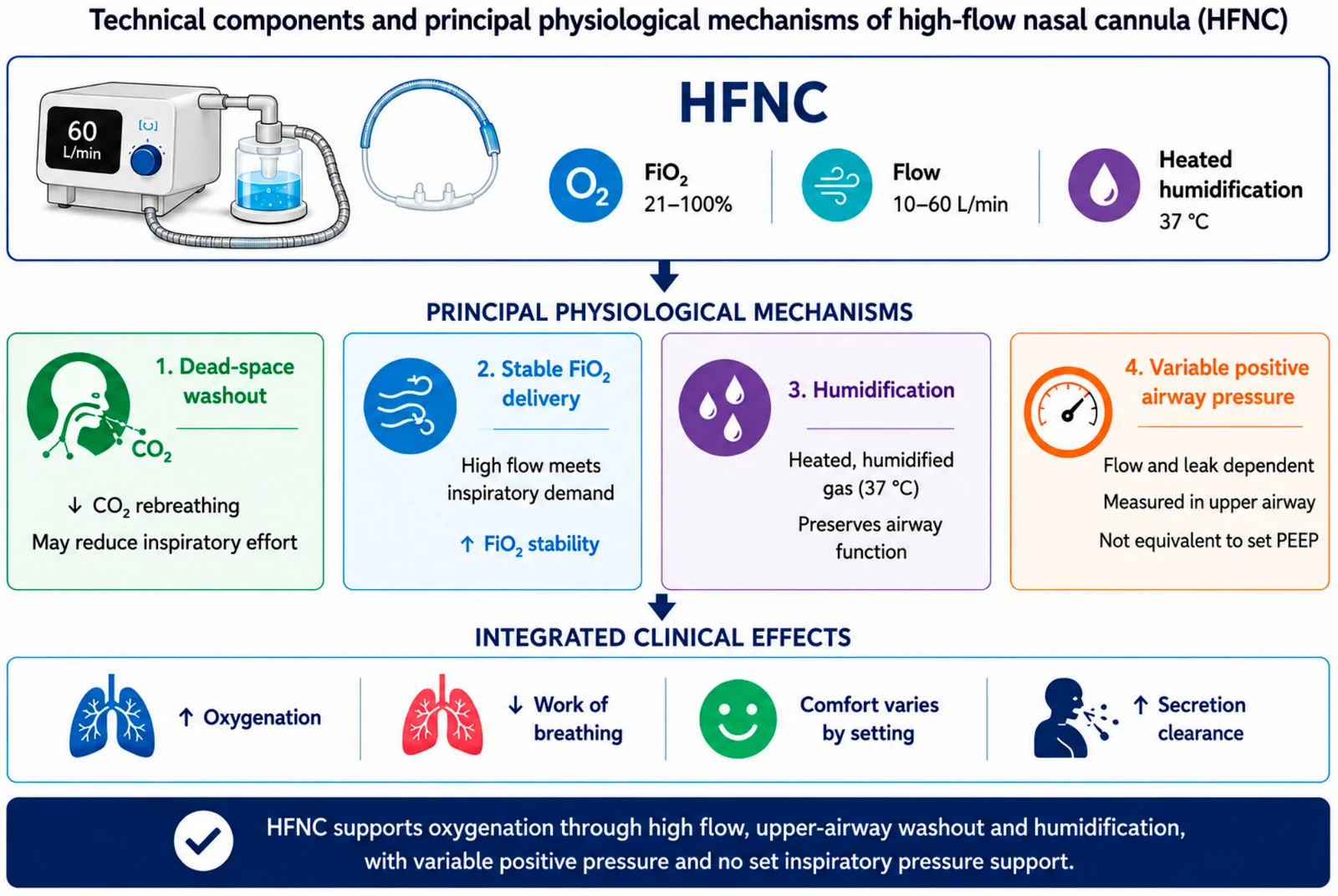
Technical delivery and principal physiological mechanisms of HFNC. Upper-airway washout, more stable inspired oxygen, humidification, and variable positive airway pressure may affect oxygenation and respiratory effort. The illustrated pressure effect is not a fixed range or prescribed PEEP. CPAP provides continuous distending pressure; bilevel NIPPV adds inspiratory pressure support. Effects depend on flow, interface leak, breathing pattern, and patient physiology [12,13,14,15].

At the same time, HFNC remains an open system. The nasal prongs do not seal the airway, and gas can escape through the mouth and/or around the cannula. Therefore, pressure generation is not controlled in the same way as with NIPPV or CPAP. The airway pressure produced by HFNC depends on delivered flow, cannula size, nostril occlusion, mouth opening, patient anatomy, and respiratory pattern [11]. This technical feature explains why HFNC may generate measurable positive airway pressure without being equivalent to pressure-based ventilatory support (Figure 1).

## 3. Physiological Effects

### 3.1. Anatomical Dead-Space Washout

The most distinctive physiological effect of HFNC is washout of upper-airway anatomical dead space. Continuous high flow clears expired CO_2_-rich gas from the nasal and pharyngeal cavities, reducing the amount rebreathed during the next inspiration [12]. This clearance increases the proportion of each breath available for effective alveolar ventilation; it does not directly remove CO_2_ from blood or guarantee a fall in PaCO_2_. Its magnitude depends on flow, interface leakage, and breathing pattern.

For a given metabolic CO_2_ production, a reduction in effective dead space can allow adequate alveolar ventilation at a lower total ventilation. Itagaki et al. used uncalibrated respiratory inductive plethysmography rather than calibrated tidal-volume measurement. They observed improved thoraco-abdominal synchrony, lower respiratory rate, and a reduction in the product of the summed plethysmography signal and respiratory rate, with stable PaCO_2_ [16]. Because accurate tidal volumes could not be obtained, this product should be interpreted as a surrogate signal, not a calibrated measurement of minute ventilation. Reduced wasted ventilation is a plausible explanation, not a directly measured result.

If fewer breaths are required to maintain CO_2_ clearance, the cumulative respiratory muscle effort per minute may decrease. A lower respiratory rate may also lengthen expiratory time. However, respiratory rate alone is not a direct measurement of work of breathing. Mauri et al. demonstrated reduced inspiratory effort using esophageal pressure measurements during HFNC in acute hypoxemic respiratory failure [13]. These findings support a reduction in respiratory load through combined mechanisms, including less rebreathing, improved oxygenation, and changes in lung volume, without implying delivery of inspiratory pressure support.

### 3.2. Positive Airway Pressure Effect

**Figure 2:**
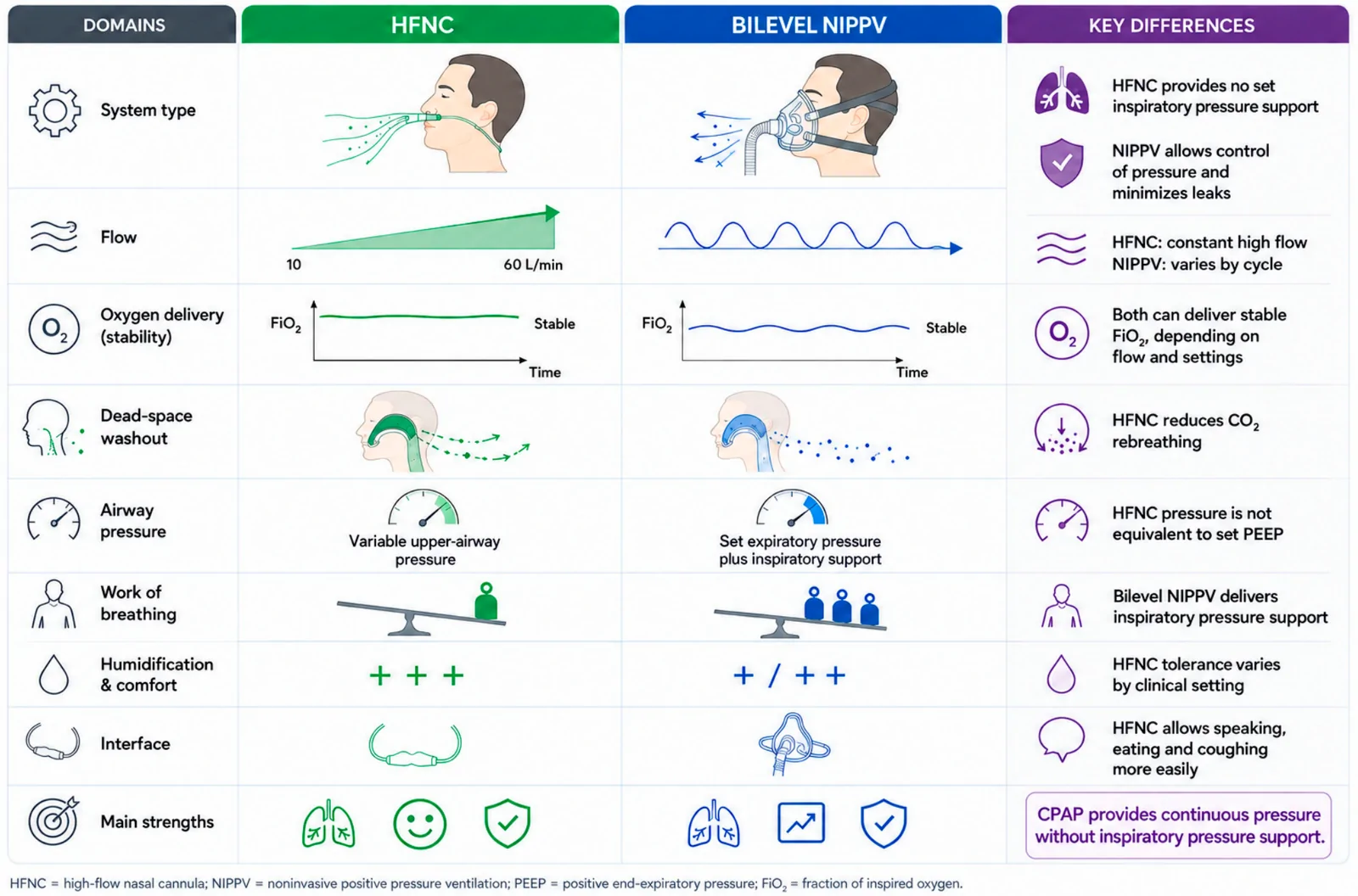
Technical and physiological comparison of HFNC and bilevel NIPPV, with clarification of CPAP. HFNC generates variable pressure measured in the upper airway; bilevel NIPPV provides prescribed expiratory pressure plus inspiratory pressure support. CPAP provides continuous distending pressure without separate inspiratory pressure support. The illustrated mechanisms do not establish clinical interchangeability [6,11,14,15].

HFNC generates variable positive airway pressure. Parke et al. measured mean nasopharyngeal pressure in postoperative cardiac surgical patients and found higher pressure with the mouth closed than open at 35 L/min [14]. Ritchie et al. reported a mean hypopharyngeal pressure of approximately 5 cmH_2_O at 50 L/min during resting nasal breathing with the mouth closed [15]. These measurements differ in site, flow, and breathing conditions; they do not support a universal 1–3 cmH_2_O range or equivalence to prescribed PEEP. Figure 1 and Figure 2 therefore describe variable, flow-dependent upper-airway pressure without a fixed numerical range.

Ritchie et al. similarly found flow-dependent increases in upper-airway pressure [15]. Lampland et al. studied an infant population; those findings should not be used to quantify adult pressure effects [17]. In adult physiological studies, pressure is commonly measured with a small catheter placed in the nasopharynx or pharynx and connected to a pressure transducer [14,15]. Such measurements describe pressure at that location, not direct tracheal or alveolar pressure, and depend on respiratory phase, mouth opening, cannula fit, and flow. Consequently, reported upper-airway pressures cannot be interpreted as a prescribed tracheal PEEP.

Corley et al. observed increased end-expiratory lung volume after cardiac surgery using electrical impedance tomography, which measures changes in end-expiratory lung impedance as a surrogate for changes in lung volume [18]. Even a small distending pressure may stabilize susceptible lung units and increase end-expiratory volume. During expiration, outward airflow interacts with the incoming high flow and the resistance of the upper airway and interface, contributing to expiratory pressure and potentially modifying lung emptying [14,15,19]. Increased expiratory resistance is therefore a possible contributor, rather than a separately quantified recruitment mechanism. An increase in end-expiratory volume does not by itself prove alveolar recruitment: depending on airway mechanics, it may also reflect altered emptying or air trapping. The respective contributions of pressure, expiratory resistance, and breathing-pattern changes remain patient-dependent.

The more accurate interpretation is that HFNC produces low-level, variable, flow-dependent pressure that may contribute to oxygenation and comfort but is insufficient to define the technique as non-invasive ventilation (Figure 2).

### 3.3. Stability of Inspired Oxygen Fraction

Another major advantage of HFNC is improved FiO_2_ stability. Wettstein et al. showed that delivered oxygen concentrations with low-flow nasal cannulas are variable and often lower than predicted, particularly during rapid breathing [20]. Ritchie et al. showed that, during nose breathing at rest, measured FiO_2_ with HFNC more closely approximated the set FiO_2_, particularly at higher flows [15].

This mechanism is clinically relevant because many patients with acute respiratory failure have high inspiratory flow demand. Conventional oxygen systems may deliver an apparently high oxygen concentration at the device level, while the patient inspires a substantially diluted mixture because of entrained room air. HFNC reduces this discrepancy by providing higher total flow and more predictable oxygen delivery.

### 3.4. Humidification and Airway Conditioning

HFNC also differs from conventional oxygen therapy because it provides heated and humidified gas. Cold and dry gas may worsen airway discomfort, impair mucociliary clearance, and increase airway resistance. Berk et al. showed that cold air can induce bronchoconstriction, while Fontanari et al. demonstrated changes in airway resistance during nasal inhalation of cold, dry, or moist air [21,22]. Salah et al. showed that nasal mucociliary transport is slower during dry-air breathing [23].

These physiological observations are often considered secondary, but they may be clinically central. Comfort, secretion clearance, and tolerance influence whether patients can remain on a therapy for prolonged periods. HFNC may therefore succeed not only because it improves gas exchange, but because patients tolerate it better than tight-fitting masks and dry oxygen systems.

## 4. Clinical Evidence

### 4.1. Acute Hypoxemic Respiratory Failure

**Figure 3:**
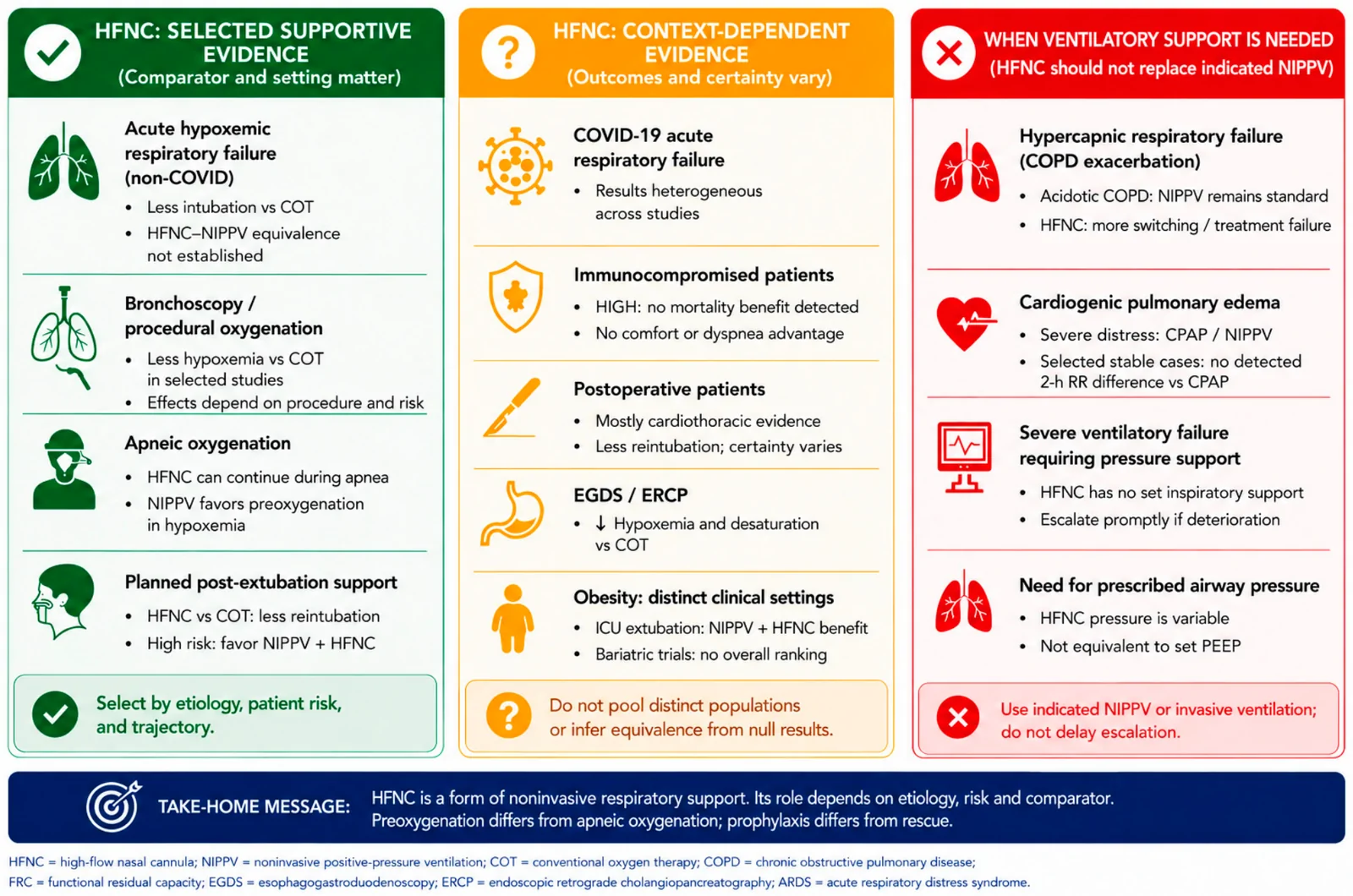
Clinical uses of HFNC organized by supportive evidence, context-dependent findings, and situations requiring ventilatory support. The colors are a narrative summary, not a formal evidence-grading framework. Comparators, risk groups, and clinical settings matter; nonsignificant comparisons do not establish equivalence. Bronchoscopy and gastrointestinal endoscopy benefits refer to comparisons with conventional oxygen therapy. High-risk planned-extubation evidence includes prophylactic NIPPV alternating with HFNC versus HFNC alone, with reintubation as the outcome. The red column cautions against replacing indicated pressure-based support; it does not exclude every selective use of HFNC. RR denotes respiratory rate. Preoxygenation is distinguished from apneic oxygenation and planned post-extubation prophylaxis from rescue. Cardiogenic pulmonary edema evidence includes a small trial in selected stable patients with no significant difference in two-hour respiratory rate change versus CPAP. Detailed outcome estimates, uncertainty, and study limitations are discussed in Section 4.1, Section 4.2, Section 4.3, Section 4.4, Section 4.5, Section 4.6, Section 4.7, Section 4.8, Section 4.9, Section 4.10 and Section 4.11.

Acute hypoxemic respiratory failure has the strongest evidence base for HFNC. Systematic reviews suggest lower intubation rates versus conventional oxygen, while mortality findings remain less consistent [24,25]. Comparisons with NIPPV vary with population, interface, and protocol; nonsignificant differences do not demonstrate equivalence [26,27]. Figure 3 summarizes clinical settings with their comparators, outcomes, and uncertainty.

The most recent and relevant evidence is the network meta-analysis by Lee et al., which included 44 randomized trials and 9704 patients with acute hypoxemic respiratory failure [28]. The authors compared CPAP, HFNC, and bilevel NIPPV with standard oxygen therapy and found that all three non-invasive respiratory supports probably reduce intubation. CPAP and HFNC may reduce mortality compared with standard oxygen therapy, but the certainty of evidence for mortality was low.

The SOHO randomized trial compared HFNC with standard oxygen in acute hypoxemic respiratory failure. Its primary outcome, 28-day mortality, was 14.6% in both groups. Intubation by day 28 occurred in 42.4% with HFNC and 48.4% with standard oxygen, but this was a secondary outcome, and the confidence intervals were not adjusted for multiplicity; the finding should therefore be considered cautiously and not considered as a definitive benefit [29]. The concept that HFNC is appropriate when oxygenation support predominates is a physiological interpretation, not a subgroup effect demonstrated by SOHO. The trial did not compare HFNC with CPAP or NIPPV.

These aggregate findings do not establish interchangeability of HFNC, CPAP, and NIPPV. In the FLORALI trial, involving nonhypercapnic patients, intubation occurred in 38% with HFNC, 47% with standard oxygen, and 50% with NIPPV; the overall comparison was not statistically significant (*p* = 0.18). However, 90-day mortality was lower with HFNC than with either comparator [30]. Excessive spontaneous effort and large tidal volumes during NIPPV are plausible contributors to patient self-inflicted lung injury (P-SILI), but FLORALI did not establish that mechanism as the cause of the mortality difference. High respiratory effort during HFNC also requires careful reassessment. Trials of de novo hypoxemic respiratory failure generally exclude admission hypercapnia and commonly exclude exacerbations of chronic respiratory disease; their results should not be extrapolated to acute alveolar hypoventilation. NIPPV remains preferred when hypercapnic ventilatory failure predominates, including in appropriate patients with COPD exacerbation, obesity hypoventilation, or neuromuscular disease. In de novo hypoxemia, persistent excessive effort, exhaustion, worsening gas exchange, or instability should prompt assessment for invasive ventilation rather than an automatic switch to NIPPV. Evidence for CPAP varies by etiology and interface and remains less extensive outside selected settings; the network estimates do not justify a universal hierarchy [6,27,28].

### 4.2. Emergency Department Acute Respiratory Failure

Tinelli et al. included four ED randomized trials comparing HFNC with conventional oxygen and one comparing HFNC with NIPPV. They detected no significant differences in intubation, treatment failure, hospitalization, or mortality; estimates were imprecise and do not establish equivalence. Discontinuation for discomfort was more frequent with HFNC than conventional oxygen, while the single NIPPV comparison did not detect differences in tolerance or dyspnea [31]. Thus, a general tolerability advantage cannot be inferred from this ED synthesis. Selection should instead reflect etiology: predominantly hypoxemic failure may justify HFNC under indication-specific guidance, acidotic COPD favors NIPPV, and cardiogenic pulmonary edema often calls for CPAP or NIPPV [6]. These distinctions are not subgroup benefits established by Tinelli et al.

HFNC can be applied rapidly in the ED, but tolerance should be assessed individually and against the relevant alternative. Persistent tachypnea, excessive effort, altered mental status, worsening gas exchange, or instability should prompt reassessment for escalation, including invasive ventilation when indicated, rather than continued HFNC based on oxygen saturation alone.

### 4.3. Viral and COVID-19 Acute Respiratory Failure

During the COVID-19 pandemic, HFNC became one of the most widely used non-invasive respiratory support strategies. Li et al. reported that HFNC reduced intubation in patients with COVID-19 acute respiratory failure, while Le Pape et al. focused on HFNC versus conventional oxygen therapy in COVID-19-related acute respiratory failure [32,33]. Carreño-Hernández et al. later synthesized evidence on non-invasive oxygenation and ventilation strategies for viral acute respiratory failure [34]. The overall interpretation is that HFNC is useful in selected patients with viral hypoxemic respiratory failure, particularly when oxygenation support and tolerance are priorities. However, COVID-19 also highlighted the danger of interpreting acceptable oxygen saturation as clinical stability. Patients may maintain oxygenation while respiratory effort remains high, potentially increasing the risk of delayed intubation or self-inflicted lung injury [35].

### 4.4. Hypercapnic Respiratory Failure

HFNC has a plausible but selective role in acute hypercapnic respiratory failure. Upper-airway dead-space washout may improve CO_2_ clearance, while humidification facilitates secretion management and the open interface often improves tolerance. Its modest positive airway pressure may partially counterbalance intrinsic PEEP in flow-limited COPD, reducing the inspiratory threshold load, although this effect is variable and cannot be titrated like external PEEP during NIPPV [36]. HFNC does not provide inspiratory pressure support. In a multicenter trial of COPD exacerbations with pH 7.25–7.35, Cortegiani et al. found that HFNC was non-inferior to NIPPV for PaCO_2_ reduction at two hours, using a 10 mm Hg non-inferiority margin, with better tolerance; nevertheless, 32% of patients assigned to HFNC switched to NIPPV by six hours [36]. Short-term non-inferiority for CO_2_ reduction does not establish equivalence for clinical outcomes.

Earlier meta-analyses did not detect clear mortality or intubation differences, but imprecision and crossover preclude equivalence [37]. Tan et al. (2024) found that initial HFNC did not meet non-inferiority for treatment failure in acidotic COPD exacerbations and produced more failures and intubations than NIPPV [38]. The composite failure outcome included intubation or switching to the other modality. Qin et al. (2025) likewise found more composite failure and treatment switching with HFNC; its nonsignificant mortality and intubation findings remained imprecise [39]. These initial-treatment comparisons differ from HFNC during NIPPV breaks or when a mask is poorly tolerated. NIPPV remains the initial standard for acidotic COPD; selective HFNC use requires clinical and blood-gas monitoring and prompt rescue for worsening acidosis or fatigue [6]. Adequately powered multicenter trials with patient-centered outcomes and prespecified rescue criteria are needed to define its selective role.

### 4.5. Cardiogenic Pulmonary Edema

In acute cardiogenic pulmonary edema, continuous distending pressure can improve oxygenation and alter cardiac loading, while bilevel NIPPV also supplies inspiratory assistance [40]. This physiological rationale should be distinguished from comparative trial findings. Altunbas et al. compared HFNC with mask-based CPAP, not bilevel pressure support, in patients with hypoxemia and increased work of breathing. Patients needing immediate intubation or with hemodynamic instability were excluded. The superiority trial detected no significant difference in respiratory rate reduction at two hours, which was its primary outcome [41].

The short observation period and crossovers limit inference about sustained clinical effectiveness, and the nonsignificant primary comparison does not establish equivalence [41]. Positive-pressure support remains an indication-specific clinical choice rather than a conclusion of this trial alone. Considering HFNC when mask intolerance limits CPAP or NIPPV, or when pressure requirements appear limited, is a clinical interpretation; a benefit restricted to milder presentations was not demonstrated as a subgroup effect.

### 4.6. Post-Extubation Support

Evidence for support following extubation concerns prophylaxis after planned extubation, which should be separated from rescue after respiratory deterioration. Fernando et al. found that NIPPV (OR 0.65, 95% CI 0.52–0.82) and HFNC (OR 0.63, 95% CI 0.45–0.87) reduced reintubation compared with conventional oxygen, with moderate certainty for both. Their NIPPV–HFNC comparison was inconclusive (OR 1.04, 95% CI 0.78–1.38; low certainty), and preventive findings were not reproduced in rescue settings [42]. In high-risk patients, Zheng et al. estimated less reintubation with NIPPV alternating with HFNC than HFNC alone (OR 0.43, 95% CI 0.28–0.65; moderate certainty); HFNC versus NIPPV alone remained inconclusive (OR 1.33, 95% CI 0.94–1.90; moderate certainty). These differing populations, combinations, and certainty assessments preclude a general equivalence claim [43]. Burns et al. evaluated spontaneous breathing trial techniques before extubation, a separate clinical question that does not establish the efficacy of support delivered afterward [44].

The ERS guideline conditionally favors prophylactic NIPPV over HFNC in patients at high risk of extubation failure unless NIPPV is contraindicated. High-risk characteristics include advanced age, chronic cardiac or respiratory disease, hypercapnia during a spontaneous breathing trial, or airway patency problems; trial criteria often include age over 65 years, but definitions vary [6]. NIPPV can alternate with HFNC between sessions to provide pressure support while retaining an open oxygenation interface. In a post hoc analysis of two trials, Thille et al. studied 829 high-risk patients without hypercapnia at extubation and found day-seven reintubation of 11.8% with NIPPV plus HFNC versus 17.6% with HFNC alone [45]. These findings do not imply that either modality should be continued as rescue therapy when reintubation is indicated.

### 4.7. Immunocompromised Patients

Azoulay et al. tested HFNC versus standard oxygen therapy in immunocompromised patients with acute respiratory failure in the HIGH randomized clinical trial [46]. The trial did not demonstrate a clear 28-day mortality benefit.

The HIGH trial reported improved oxygenation with HFNC but did not detect significant differences in comfort or dyspnea compared with standard oxygen; its secondary analyses were exploratory [46]. A general tolerability rationale for the open HFNC interface should therefore not be presented as a benefit established in immunocompromised patients. The absence of a significant 28-day mortality difference does not establish clinical equivalence.

### 4.8. Preoxygenation and Peri-Intubation Management

For preoxygenation before induction in acute hypoxemic respiratory failure, Fong et al. found a higher lowest SpO_2_ during intubation with NIPPV than HFNC (mean difference 3.58 percentage points, 95% CI 0.59–6.57) [47]. This comparative finding should be separated from the proposed mechanism of recruitment with positive pressure. Selection based on severe shunt, respiratory effort, or obesity reflects physiological reasoning rather than a differential treatment effect established by this network analysis. HFNC maintained during laryngoscopy provides apneic oxygenation, a distinct use from preoxygenation before induction.

A complementary strategy is to use NIPPV for preoxygenation before induction, with HFNC positioned underneath the mask and maintained during apnea and laryngoscopy after mask removal. The small randomized OPTINIV trial evaluated this combination and found improved peri-intubation oxygenation compared with NIPPV alone [48]. This approach combines pressure-based preoxygenation with continued apneic oxygen delivery; it should not delay intubation or be assumed to provide ventilation or adequate CO_2_ clearance during apnea.

### 4.9. Postoperative Patients and Obesity

Postoperative prophylaxis differs from support after prolonged ICU ventilation. Lu et al. summarized postoperative HFNC comparisons with conventional oxygen [49]. In the predominantly cardiothoracic evidence synthesized by Chaudhuri et al., HFNC reduced reintubation versus conventional oxygen with moderate certainty, while certainty for reduced escalation of respiratory support was very low [50]. The apparent concentration of benefit in obese or high-risk patients came from post hoc subgroup analysis and should be viewed as hypothesis-generating. The limited HFNC–NIPPV comparison did not establish equivalence.

In critically ill adults with obesity after extubation, Pensier et al. found reduced reintubation with NIPPV alternating with HFNC compared with HFNC alone, whereas the estimate for NIPPV alone versus HFNC was imprecise [51]. Separately, Li et al. studied postoperative obese patients, largely in bariatric surgical settings. Bilevel support and HFNC reduced atelectasis versus conventional oxygen, while differences in pneumonia, hypoxemia, respiratory failure, and oxygen-treatment failure were not significant. The authors reported better oxygenation with CPAP and shorter hospital stay with HFNC, whereas nonsignificant comparisons for other outcomes and SUCRA rankings do not prove overall superiority [52]. Reduced functional residual capacity and recruitment requirements provide physiological context, not a universal mandate for NIPPV or CPAP across these distinct populations.

### 4.10. Procedural Sedation, Endoscopy, and Bronchoscopy

HFNC has reduced hypoxemia compared with conventional oxygen in selected gastrointestinal endoscopy, bronchoscopy, and procedural sedation studies [53,54,55,56,57]. The magnitude of benefit depends on the procedure, baseline risk, sedation, and oxygen comparator. These procedural comparisons should be distinguished from preoxygenation before induction and from apneic oxygenation during laryngoscopy. HFNC does not provide controlled ventilation; improved saturation does not exclude hypoventilation, and ventilation monitoring remains necessary when indicated.

### 4.11. Extracorporeal Membrane Oxygenation

HFNC may be used as an adjunctive oxygenation strategy during awake or extubated VV-ECMO, mainly to maintain upper-airway oxygen delivery, improve comfort, and facilitate avoidance of invasive ventilation [58]. However, available evidence is mainly observational and derives from broader awake-ECMO strategies rather than from studies specifically designed to evaluate HFNC itself. Observational cohorts, case series, and narrative reviews suggest that HFNC may facilitate spontaneous breathing, communication, and mobilization during awake VV-ECMO, but no robust comparative evidence demonstrates improved patient-centered outcomes attributable specifically to HFNC [59,60,61].

## 5. Complications and Safety

HFNC is generally well tolerated. Common adverse effects include nasal discomfort, abdominal distension, and dryness when humidification is inadequate. Lazovic et al. reviewed unusual complications of NIPPV and HFNC, including rare reports of barotrauma [62].

The main safety concern is delayed recognition of treatment failure. Oxygen saturation may improve despite persistent high respiratory effort [63]. Monitoring should include respiratory rate, accessory muscle use, mental status, gas exchange, hemodynamics, and their trajectory. The ROX index is calculated as (SpO_2_/FiO_2_)/respiratory rate, with SpO_2_ expressed as a percentage and FiO_2_ as a fraction. In the prospective validation by Roca et al. in pneumonia-related hypoxemic respiratory failure, ROX ≥ 4.88 at 2, 6, or 12 h was associated with a lower risk of intubation, whereas values below 2.85, 3.47, and 3.85 at those respective times identified higher risk [64]. Serial assessment can support early recognition of failure, but thresholds depend on population and timing. ROX is an adjunct to bedside judgment, not a stand-alone intubation rule or a reason to wait when deterioration is already evident; it does not assess hypercapnic acidosis or directly measure respiratory muscle fatigue.

## 6. Common Misconceptions About HFNC

The first misconception is that HFNC is a form of NIPPV. HFNC is an open high-flow oxygen system, whereas NIPPV is a pressure-targeted ventilatory support strategy [65].

The second misconception is that the positive airway pressure generated by HFNC is equivalent to set PEEP. Its modest distending pressure may have clinically relevant effects on lung volume and inspiratory threshold load in selected patients, but it remains variable and depends on flow, mouth closure, cannula fit, and anatomy [14,19]. Upper-airway pressure measurements should not be equated with controlled tracheal PEEP.

The third misconception is that improved SpO_2_ means respiratory failure is resolving. Oxygenation can improve while respiratory effort remains excessive [35].

This discordance does not mean that HFNC intrinsically conceals respiratory failure. Rather, clinicians may be falsely reassured if they assess oxygen saturation without considering respiratory effort and the overall clinical trajectory. Serial ROX assessment can help identify patients at risk of failure, alongside examination and blood-gas monitoring when indicated [64].

The fourth misconception is that reduction in intubation implies mortality benefit or equivalence to NIPPV [66]. The recent network meta-analysis by Lee et al. suggests that HFNC may reduce mortality compared with standard oxygen therapy, but certainty is low and the finding should not be extrapolated to all populations or interpreted as physiological equivalence to NIPPV [28].

## 7. Limitations of This Review

This is a narrative, physiology-oriented review, not a systematic review or guideline. We did not undertake a protocol-based comprehensive search, duplicate screening, formal risk-of-bias assessment, or quantitative synthesis. For this revision, targeted identification and verification of the literature used PubMed-indexed records and journal websites, with the latest targeted update on 5 October 2026. Topic-focused queries combined HFNC or high-flow nasal oxygen with physiological mechanisms and acute-care indications, and reviewer-suggested studies were checked against their source reports. We prioritized guidelines and systematic or network reviews for aggregated outcomes and primary physiological or randomized studies for mechanisms, measurement methods, and pivotal or newer comparisons. This targeted update does not establish exhaustive coverage; selection remains narrative and may be subject to selection and interpretation bias.

## 8. Conclusions

HFNC is an appropriate initial option for selected patients with predominantly hypoxemic failure, but its open delivery differs from CPAP and NIPPV. Evidence for initial use in acidotic COPD does not support routine replacement of NIPPV; selected use during breaks or intolerance is a separate question. Early reassessment should consider respiratory rate and effort, oxygen requirements, gas exchange, mental status, and hemodynamics, with serial ROX assessment when appropriate. A review within roughly 1–2 h may be a pragmatic bedside checkpoint, not a validated failure threshold. Deterioration or an immediate indication for intubation requires action without waiting for that interval. Escalation should follow etiology and trajectory and must not delay invasive ventilation when needed.

## Data Availability

The original contributions presented in this study are included in the article. Further inquiries can be directed to the corresponding author.

## Abbreviations

The following abbreviations are used in this manuscript:

ARDS	Acute respiratory distress syndrome
COPD	Chronic obstructive pulmonary disease
COT	Conventional oxygen therapy
CPAP	Continuous positive airway pressure
ECMO	Extracorporeal membrane oxygenation
ED	Emergency department
EGDS	Esophagogastroduodenoscopy
ERCP	Endoscopic retrograde cholangiopancreatography
FiO_2_	Fraction of inspired oxygen
FRC	Functional residual capacity
HFNC	High-flow nasal cannula
ICU	Intensive care unit
NIPPV	Non-invasive positive-pressure ventilation
PaCO_2_	Arterial partial pressure of carbon dioxide
PEEP	Positive end-expiratory pressure
SpO_2_	Peripheral oxygen saturation
VV-ECMO	Veno-venous extracorporeal membrane oxygenation
NIRS	Non-invasive respiratory support
P-SILI	Patient self-inflicted lung injury
ROX	Ratio of oxygen saturation to inspired oxygen fraction divided by respiratory rate
